# Global Fund financing and human resources for health investments in the Eastern Mediterranean Region

**DOI:** 10.1186/s12960-020-00483-x

**Published:** 2020-07-08

**Authors:** Adeyemi Okunogbe, Diana Bowser, Gulin Gedik, Saha Naseri, Ayat Abu-Agla, Najibullah Safi

**Affiliations:** 1 Global Health Division, RTI International, Washington, DC USA; 2 Heller School for Social Policy and Management, Brandeis University, 415 South Street, Waltham, MA 02453 USA; 3 Health Workforce, Regional Office for Eastern Mediterranean, World Health Organization, Cairo, Egypt; 4 World Health Organization Afghanistan Country Office, Kabul, Afghanistan; 5 World Health Organization Sudan Office, Khartoum, Sudan

**Keywords:** Global Fund, Human resources for health, Eastern Mediterranean Region, Budget analysis, Health system strengthening

## Abstract

**Background:**

Despite the large investments in donor-related health activities in areas of the globe prone to tension and conflict, few studies have examined in detail the role of these donor investments in human resources for health (HRH).

**Methods:**

We used a mixed-methods research methodology comprising both quantitative and qualitative analyses to analyze the Enhanced Financial Reporting System of the Global Fund to Fight AIDS, Tuberculosis and Malaria budget and expenditure data from 2003 to 2017 for 13 countries in the Eastern Mediterranean Region (EMR). We analyzed additional detailed budgetary data over the period 2015–2017 for a sub-set of these countries. Two country-case studies were conducted in Afghanistan and Sudan for a more in-depth understanding of the HRH-related activities that occurred as a result of Global Fund grants.

**Results:**

The results show that US$2.2 billion Global Fund dollars had been budgeted and US$1.6 billion were expended over the period 2003–2017 in 13 Eastern Mediterranean countries. The average expenditures for human resources for health (training and human resources) as a percentage of total expenditure are 28%. Additional detailed budgetary data analysis shows a more conservative investment in HRH with 13% of total budgets allocated to “direct” HRH activities such as salaries, training costs, and technical assistance. HRH-related activities supported by the Global Fund in Afghanistan and Sudan were similar, including pre-service and in-services training, hiring of program coordinators and staff, and top-ups for clinical staff.

**Conclusions:**

HRH remains a key issue in strengthening the health systems of low- and middle-income countries. While this study suggests that Global Fund’s HRH investments in the EMR are not lagging behind the global average, there appears to be a need to further scale up these investments considering this region’s unique HRH challenges.

## Introduction

The Global Fund to Fight AIDS, Tuberculosis and Malaria (the Global Fund) was founded in 2002 to accelerate the end of the epidemics of these devastating diseases in low- and middle-income countries (LMICs) [1]. The organization also seeks to strengthen health systems in countries through direct health system strengthening investments and indirectly through investing in interventions across the three diseases. An important area of investment is human resources for health (HRH), which the World Health Organization describes as one of the six core components or “building blocks” of health systems [2]. Many LMICs, especially those in areas with recent conflicts and instability, face critical HRH challenges including health worker shortage, geographic maldistribution and migration, skill-mix imbalance, weak regulation, poor work environment, and poor quality and limited capacity of educational and training programs [3–6]. Increasingly, it is acknowledged that the health workforce availability and quality are critical in the implementation of externally funded projects, such as those funded by the Global Fund.

Since its inception in 2003, the Global Fund has invested over US$40 billion in over 100 countries to combat human immunodeficiency virus/acquired immunodeficiency syndrome (HIV/AIDS), tuberculosis (TB), and malaria [1], with a certain percentage of this allocated to HRH-related activities, depending on the country and its needs. A study of 138 Global Fund recipient countries estimated that around US$1.4 billion (23% of total US$6.2 billion) was allocated to HRH-related activities between the first and seventh round of funding over the period 2003–2008 [7]. Global Fund investments strengthened health workforce in recipient countries through funding short-term and in-service training, as well as innovative remuneration of health workers [7–10], though investment in HRH was mainly limited to in-service training and supporting program management staff [8]. Other studies suggest potential unintended negative consequences of disease-focused investments in human resources through the displacement of health workers to funded programs to the detriment of adequately staffing other health programs [11–13].

This study builds off the limited existing research on how HRH has been influenced by donor investments in LMICs [7]. The aim of the study is to examine the specific role of the Global Fund in strengthening HRH in the Eastern Mediterranean Region (EMR). The EMR in this context are 21 countries and territories served by the World Health Organization (WHO) Regional Office in the Eastern Mediterranean. This region, prone to tension and conflict, has one of the lower overall HRH densities among the six WHO regional groupings [4, 5, 14]. As of 2018, 8% of total investments by the Global Fund have been in North Africa and the Middle East, the third largest behind Sub-Saharan Africa (65%) and Asia and the Pacific (19%) [15]. In addition, there has been minimal investigation of the role of such investments in improving HRH in this region. Our research questions are as follows: (1) What are the levels and composition of Global Fund investments in HRH in EMR countries? (2) What types of HRH activities have been supported by these investments? and (3) In what ways have these investments contributed to health system strengthening in some of these countries?

To answer these questions, we examine the Global Fund financing for HRH across those EMR countries that received Global Fund grants. We then focus on two of these countries in more detail to understand the types of HRH activities supported and contributions to health system strengthening. This study adds to the policy dialogue on the role of global health initiatives in improving HRH, and by extension health systems in LMICs, especially in the EMR.

## Methods

**Fig. 1:**
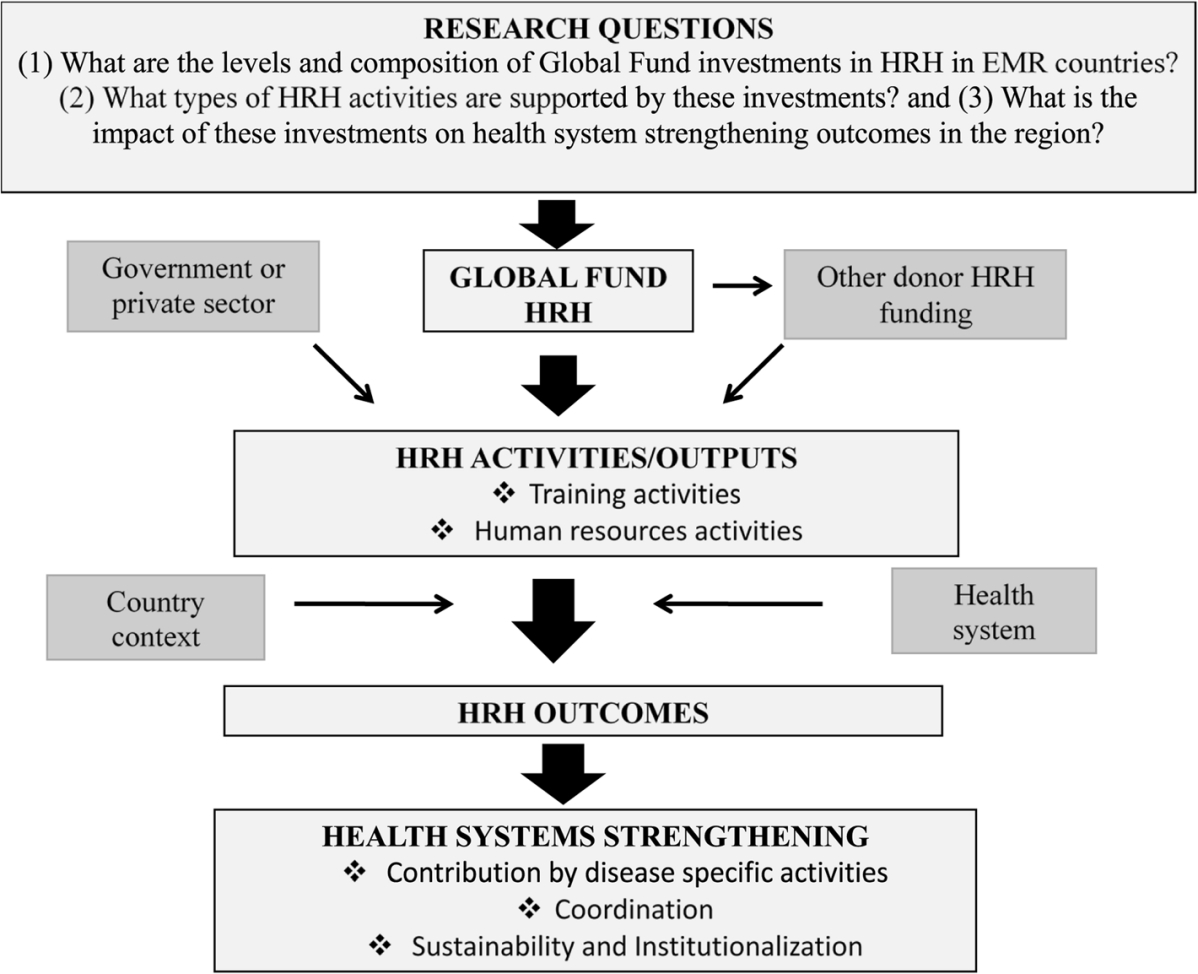
Analytical framework. Notes: HRH denotes human resources for health; EMR denotes Eastern Mediterranean Region

The analytical framework used in this study, as shown in Fig. 1, was adapted to the EMR and was used to guide the methods and analysis for this research [7]. The magnitude of Global Fund HRH investments in EMR countries over the period 2003–2017 was tracked and captured. Global Fund investments were linked to specific HRH activities which were in turn associated with potential HRH outcomes and health systems strengthening.

**Fig. 2:**
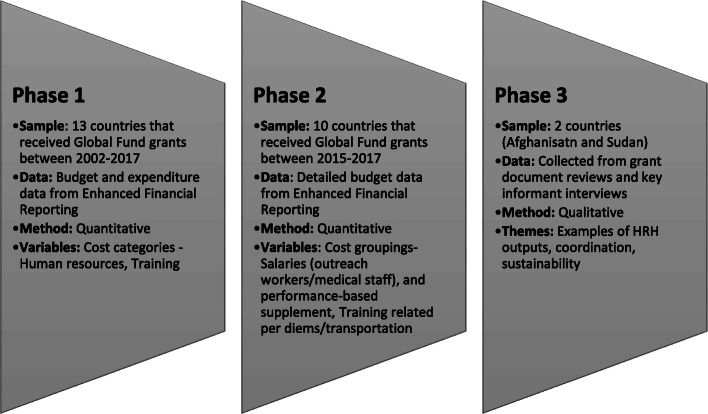
Phases of the analysis. Notes: HRH denotes human resources for health

We employed mixed research methodology comprising both quantitative and qualitative analyses in three main phases as illustrated in Fig. 2 [16]. Phases 1 and 2 involved utilizing quantitative methods to examine the magnitude of HRH investments as well as compositions by income group, disease focus, and Global Fund regional team categorizations. The results from phases 1 and 2 were then complemented in phase 3 with case studies of selected countries in the region (Afghanistan and Sudan). The key outputs examined were training- and human resources (HR)-related outputs, such as HR financing (salary support, performance incentives, for example), hiring, and recruitment [7].

In phase 1, we analyzed budgetary and expenditure data for the 13 EMR countries that had received Global Fund grants using data from the Global Fund Enhanced Financial Reporting System over the period 2003 (Global Fund inception) to 2017 (the last year of complete data before analysis was conducted). These countries were Afghanistan, Djibouti, Egypt, Iran, Iraq, Jordan, Morocco, Pakistan, Somalia, Sudan, Syrian Arab Republic, Tunisia, and Yemen. This analysis examined the total aggregate Global Fund investments in each country and lower-level investments in human resources, training activities, and technical assistance. We also analyzed the compositions of these investments by disease categories (HIV/AIDS, TB and malaria), by income groupings (based on 2018 World Bank income classifications) [17] and Global Fund regional team categorizations in the region. The budgetary and expenditure data provided high-level aggregate summaries for the four main cost categories: human resources, training, technical assistance, and “other”. “Other” category was comprised of non-HRH-related cost categories such as “medicines and pharmaceutical products,” “monitoring and evaluation,” “overheads,” “planning and administration,” “procurement and supply management costs,” “infrastructure and other equipment,” and “health products and health equipment.” These categories were based on Global Fund reporting requirements and guidelines [18] and were a result of high-level aggregates across the funding models they used, namely the round-based system (2003-2013) and the new funding model (2014 until current date). It was not possible, due to data limitations within the Global Fund reporting system, to obtain disaggregated and more detailed data within these high-level aggregates over the period of 2003–2013. With the new funding model, data available after 2015 did incorporate more detailed and disaggregated cost categories. Total amounts budgeted and expensed for training and human resources were examined as well as the percent of these amounts in comparison to total Global Fund budgets and expenses across the years of funding. These percentages were also examined over time, using the time periods 2003–2007 and 2008–2017 to compare to a similar analysis done prior to 2007 [7].

For this reason, in phase 2, we tracked and documented spending within the cost categories using more detailed budgetary data acquired from the Global Fund over period 2015–2017. This data set provided budgetary sub-categories (cost groupings) for three main HRH-related cost categories: human resources, travel-related costs (TRC) formerly referred to as training, and external professional services formerly referred to as technical assistance. The cost groupings under these cost categories were as follows (Table 1): *human resources*: salaries (program management), salaries (outreach workers/medical staff), and performance-based supplement, and other human resources costs; *travel-related costs (TRC)*: training-related per diems/transportation/other costs, technical assistance-related per diems/transportation/other costs, supervision/survey/data collection-related per diems/transportation costs, meeting/advocacy-related per diems/transportation/other costs, and other transportation costs; and *external professional services*: technical assistance fees/consultants, fiscal/fiduciary agent fees, external audit fees, and other external professional services.

**Table 1:** Cost categories and cost groupings/sub-categories

**Human resources**	**Training or travel-related costs**	**Technical assistance or external professional services**
**Direct HRH allocations**		
Salaries (outreach workers/medical staff)	Training-related per diems/transport/other costs	Technical assistance fees-consultants
Performance-based supplement, incentives		
**Other allocations not directly related to HRH**		
Salaries (program management)	Technical assistance-related per diems/transport/other costs	Fiscal/fiduciary agent fees
Other human resources costs	Supervision/surveys/data collection related per diems/transport/other costs	External audit fees
	Meeting/advocacy-related per diems/transport/other costs	
	Other transportation costs	

*HRH* human resources for health

All other sub-categories or cost groupings which were not direct investments in local human resources for health in a country were classified as “indirect” HRH costs. For example, under human resources, the sub-category designated as salaries (program management) captured funds allocated by Global Fund to pay salaries for the managers within organizations (principal recipients) that administered the grants. These allocations were not direct investments in health cadres that provided clinical services and hence were not considered direct human resource strengthening investments for the purpose of this study.

According to the framework of this study, direct investment in HRH was captured through allocations that most directly impacted the hiring and training of service providers (clinical cadres). We identified cost groupings within human resources, training, and technical assistance that most clearly overlapped with hiring and training. These were called direct HRH financial investments in this study (Table 1). These direct financial investments were those in the top panel in Table 1: “salaries (outreach workers/medical staff), and performance-based supplement” [under human resources]; “training-related per diems/transportation/other costs” [under TRC], and “technical assistance fees/consultants” [under external professional services /technical assistance]. We calculated the percent of the total budget as well as the percent of each cost category (human resources, TRC, and external professional services/technical assistance) that were allocated to these direct HRH investments. These percentages were referred to as direct HRH budget allocations and served as lower bound estimates within the upper bound estimates that captured the total proportion of Global Fund HRH investments in human resources and training.

In phase 3, two countries—Afghanistan and Sudan—were selected from the region for case studies, taking into consideration the variation in Global Fund financing for human resources, density of human resources, and feasibility of conducting key informant interviews across the 21 EMR countries. The case study methodology involved a desk review of Global Fund grant proposals and performance reports as well as sending an interview form to agencies and programs that had been involved in the Global Fund programs in each country. The interview form collected qualitative information on training and human resource investments made with Global Fund financing. Follow-up meetings and/or phone calls were held with focal points from the agencies and programs to verify information from the interview form. All responses were voluntary, and respondent answers were de-identified for the analysis to ensure confidentiality and anonymity. A total of 14 focal points were interviewed from 10 key agencies/programs in Afghanistan and Sudan. The case study results focused on activities related to grants that were active between 2015 and 2017.

## Results

A total of 13 countries from the Eastern Mediterranean Region who were previous and current grant recipients are included in this study. Of these 13 countries, nine were funded during the study period. Four countries were not funded as of 2018, but had received grants in the past. About half of these countries were in the World Bank lower-middle-income category (Table 2) [17]. With respect to HRH density, Global Fund recipient countries have physician and nurse/midwife HRH densities below the regional average in the EMR region, based on the most recent data available.

**Table 2:** Global Fund-recipient countries and human resources for health data

Country	1st year of Global Fund grant	Most recent year of Global Fund grant	Physicians per 1000 pop. (year)^**a**^ [pre funding]	Physicians per 1000 pop. (year)^**a**^ [most recent]	Nurses/midwives per 1000 pop. (year)^**b**^ [pre funding]	Nurses/midwives per 1000 pop. (year)^**b**^ (most recent)	HR/training investment as percentage of total expenditure^**c**^	Region^**d**^	Income^**e**^	HSS/RSSH grant^**f**^
Afghanistan	2004	2017	0.20 (2001)	0.28 (2016)	0.60 (2005)	0.32 (2017)	33.4%	SEA	Low-income	Yes
Djibouti	2007	2017	0.23 (2006)	0.22 (2014)	0.57 (2005)	0.54 (2014)	33.5%	MENA	Lower-middle-income	No
Egypt	2004	2016	0.52 (2003)	0.79 (2017)	1.95 (2004)	1.40 (2017)	38.1%	MENA	Lower-middle-income	No
Iran	2005	2018	0.87 (2004)	1.14 (2015)	1.38 (2004)	1.87 (2015)	34.5%	SEA	Upper-middle-income	No
Iraq	2008	2017	0.64 (2010)	0.82 (2017)	No data	1.68 (2017)	27.4%	MENA	Upper-middle-income	No
Jordan	2003	2014	2.22 (2002)	2.34 (2017)	2.84 (2002)	3.39 (2017)	30.5%	MENA	Upper-middle-income	No
Morocco	2007	2017	0.53 (2004)	0.73 (2017)	0.81 (2004)	1.10 (2017)	33.6%	MENA	Lower-middle-income	Yes
Pakistan	2004	2018	0.68 (2001)	0.98 (2015)	0.44 (2001)	0.50 (2015)	29.4%	HIA	Lower-middle-income	Yes
Somalia	2004	2017	0.03 (2006)	0.02 (2014)	0.09 (2006)	0.06 (2014)	34.0%	MENA	Low-income	No
Sudan	2005	2018	0.25 (2004)	0.41 (2015)	1.04 (2004)	0.83 (2015)	20.9%	MENA	Lower-middle-income	Yes
Syrian Arab Republic	2007	2016	1.54 (2005)	1.22 (2016)	1.89 (2005)	1.46 (2016)	24.5%	MENA	Low-income	No
Tunisia	2007	2017	0.93 (2005)	1.27 (2016)	2.85 (2004)	2.64 (2016)	40.7%	MENA	Lower-middle-income	No
Yemen	2004	2016	0.34 (2004)	0.31 (2014)	0.69 (2004)	0.73 (2014)	16.2%	MENA	Low-income	No
Average (range for all 21 EMR countries)	2003–2008	2014–2018	1.08 (0.03–3.42)	1.20 (0.001–2.58)	2.09 (0.09–5.64)	2.74 (0.06–3.39)	28.0%			

*HSS/RSSH* health systems strengthening/resilient and sustainable systems for health

^a^Total number of physicians per 1000 population from WHO HRH workforce database and EMR Health Observatory for year with available data most proximate to funding year [19]

^b^Total number of nurses/midwives per 1000 population from WHO HRH workforce database and EMR Health Observatory for year with available data most proximate to funding year [19]

^c^Based on authors’ calculations, expenditures on HR, and training activities as a share of total expenditures from 2002–2017 as categorized by the Global Fund’s Enhanced Reporting Framework

^d^Global Fund regional team groupings: *SEA* South East Asia, *MENA* Middle East and North Africa, *HIA* High Impact Asia

^e^World Bank income-level classification (2018) [17]

^f^Health systems strengthening (HSS) or resilient and sustainable systems for health (RSSH) grant awarded by the Global Fund

### Quantitative findings

**Fig. 3:**
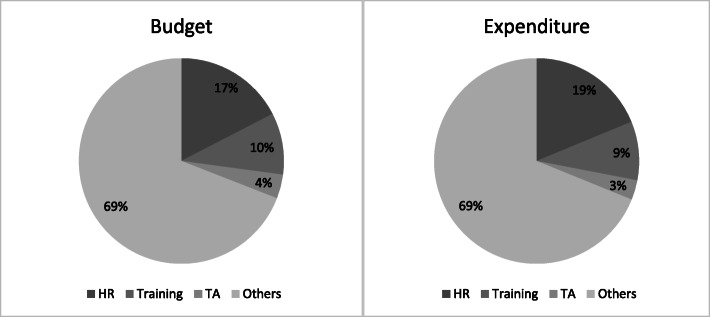
Human resource/training as share of total budget and expenditure for the 13 recipient countries, 2003–2017. Notes: HR denotes human resources; TA denotes technical assistance

As shown in Table 3, based on the quantitative analysis of the 13 grant recipients that received Global Fund funding at some point over the period 2003–2017, we estimate about US$2.2 billion in budgeted funding from Global Fund grants and US$1.6 billion in Global Fund grant expenditure. Budgetary allocations to human resources for health (training and human resources) as a percentage of total budget range from 15% in Yemen to 35% in Tunisia. Similarly, actual expenditures as a percentage of total expenditure range from 17% in Yemen to 40% in Tunisia. Figure 3 shows that budgetary allocations to and spending on human resources for health (human resources and training combined) are 27% of total budget (US$599 million) and 28% of total expenditure (US$454 million), respectively. We also examine the percentages allocated to training and human resources combined pre- and post-2007. While an average of 21% and 23% of total budgets and expenditure from the Global Fund were allocated to HRH in the EMR from 2003 to 2007 respectively, about 28% of both total budgets and expenditure from the Global Fund were allocated between 2008 and 2017 (Additional file 1: Table S2).

**Table 3:** Budgetary allocation and expenditure: total, human resources (HR), training, and technical assistance (TA), 2003–2017 [17, 19]

	Budget, US$ (%*)				Expenditure, US$ (%*)			
Country	Total	HR	Training	TA	Total	HR	Training	TA
Afghanistan	233 194 027	52 778 437 (23)	20 239 644 (9)	10 455 030 (4)	152 381 656	39 801 938 (26)	11 095 320 (7)	6 641 728 (4)
Djibouti	42 113 881	8 628 152 (20)	2 868 552 (7)	5 683 562 (13)	29 538 718	8 402 271 (28)	1 480 063 (5)	3 523 896 (12)
Egypt	36 249 650	4 098 166 (11)	6 762 898 (19)	873 209 (2)	20 682 336	3 070 078 (15)	4 802 987 (23)	573 123 (3)
Iran	105 864 066	27 264 417 (26)	10 057 123 (10)	2 426 481 (2)	88 989 207	23 171 534 (26)	7 531 365 (8)	2 159 921 (2)
Iraq	46 105 079	7 136 445 (15)	4 955 563 (11)	2 574 817 (6)	37 622 702	5 960 003 (16)	4 331 434 (12)	2 270 294 (6)
Jordan	11 829 492	680 380 (6)	2 599 742 (22)	667 250 (6)	10 024 190	644 986 (6)	2 412 680 (24)	329 404 (3)
Morocco	77 363 875	14 738 094 (19)	9 812 319 (13)	2 761 777 (4)	59 487 248	12 246 258 (21)	7 715 850 (13)	1 434 215 (2)
Pakistan	574 114 044	115 499 162 (20)	45 424 729 (8)	18 876 889 (3)	400 128 645	87 932 912 (22)	29 555 818 (7)	9 924 518 (2)
Somalia	284 468 763	70 311 538 (25)	19 558 873 (7)	11 276 351 (4)	228 025 183	59 777 440 (26)	17 670 720 (8)	8 510 901 (4)
Sudan	646 996 466	65 699 622 (10)	78 739 948 (12)	19 153 873 (3)	490 722 982	48 303 597 (10)	54 474 173 (11)	9 509 023 (2)
Syrian Arab Republic	12 497 847	1 460 081 (12)	1 231 581 (10)	302 779 (2)	8 434 865	1 078 228 (13)	991 562 (12)	49 207 (1)
Tunisia	40 002 624	8 688 370 (22)	5 482 207 (14)	2 434 255 (6)	25 216 896	6 405 462 (25)	3 870 355 (15)	979 853 (4)
Yemen	97 849 659	7 849 150 (8)	6 685 193 (7)	5 941 158 (6)	66 785 277	7 031 476 (11)	3 768 115 (6)	4 221 680 (6)
**Total**	**2 208 649 474**	**384 832 013 (17)**	**214 418 371 (10)**	**83 427 432 (4)**	**1 618 039 903**	**303 826 183 (19)**	**149 700 442 (9)**	**50 127 765 (3)**

*Percentage of total Global Fund budget or expenditure for the country (row) as applicable

**Fig. 4:**
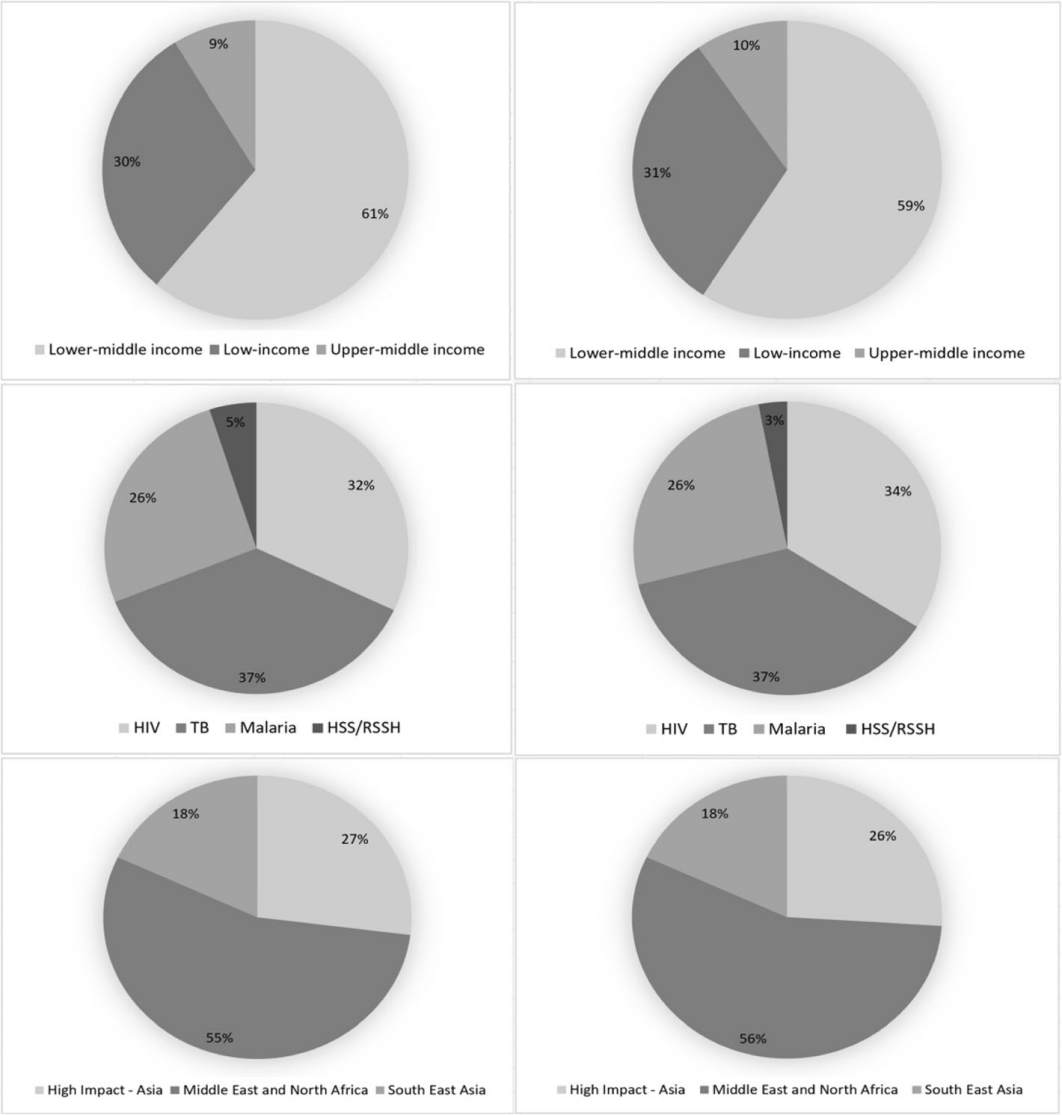
Human resource/training as share of total budget and expenditure to human resource/training for the 13 recipient countries, by income classification, disease component, and Global Fund regions, 2003–2017. Notes: HIV denotes human immunodeficiency virus; TB denotes tuberculosis; HSS/RSSH denotes health systems strengthening/resilient and sustainable systems for health; Three left hand charts reflect budgets while the three right hand charts reflect expenditures

Analysis of the total amount of human resources/training budget and expenditure allocated by income level, disease category, and Global Fund region is shown in Fig. 4. As shown, about 60% of the total human resources/training budget and expenditure is allocated to HR/training in lower-middle-income countries, probably reflecting the fact that about half of the countries in our analysis are in the lower-middle-income category. We find that about 37% of total HR/training allocation in the study countries is for TB, while 26% is for malaria. About 32% of total HR/training budget and 34% of total HR/training expenditure are for HIV/AIDS. Some grants are designated entirely for health system strengthening activities. About 5% of total budget allocation to HR/training and 3% of total HR/training expenditure across all grants from 2003 to 2017 are within the health systems strengthening/resilient and sustainable systems for health (HSS/RSSH) component.

Using the direct estimates of HRH (HR and training) budgetary allocation from the detailed budget data from 2015 to 2017, we show that 10 countries in the region are recipients of Global Fund grants within this 3-year period. As shown in Table 4, 36% of total grants’ budgets from the Global Fund are allocated to HR and training (or TRC) between 2015 and 2017. However, analysis of only the line items that are specific to direct investments to local health workers (e.g., excluding indirect allocations such as payment to grant management workers ) shows that 13% of total budget is allocated to direct HRH (HR and training).

**Table 4:** Global Fund grant budgets, 2015–2017, US dollars

		Human resources (HR)				Training or travel-related costs					
Country	Total budget	Total HR	Total HR as percent of total budget	Direct HR	Direct HR as percent of total budget	Total training	Total training as percent of total budget	Direct training	Direct training as percent of total budget	(HR + training) as percent of total budget	Direct (HR + training) as percent of total budget
Afghanistan	54 126 807	17 167 474	32%	4 234 575	8%	7 959 313	15%	3 309 321	6%	46%	14%
Djibouti	16 432 878	3 155 907	19%	1 307 876	8%	2 165 779	13%	598 829	4%	32%	12%
Iran	14 007 345	5 178 361	37%	3 089 783	22%	722 675	5%	408 198	3%	42%	25%
Iraq	3 000 000	739 249	25%	386 000	13%	487 665	16%	38 800	1%	41%	14%
Morocco	6 320 970	281 006	4%	65 048	1%	1 249 151	20%	714 252	11%	24%	12%
Pakistan	193 601 866	46 370 112	24%	11 773 393	6%	23 447 800	12%	5 653 144	3%	36%	9%
Sudan	177 988 894	17 695 567	10%	4 997 090	3%	36 161 698	20%	13 489 787	8%	30%	10%
Somalia	79 411 878	24 678 753	31%	16 612 437	21%	12 770 065	16%	4 782 743	6%	47%	27%
Tunisia	4 343 479	960 956	22%	485 403	11%	431 697	10%	339 866	8%	32%	19%
Yemen	19 643 701	438 010	2%	–	0%	3 372 863	17%	1 244 367	6%	19%	6%
**Total/mean**	**568 877 818**	**116 665 396**	**21%**	**42 951 605**	**8%**	**88 768 705**	**16%**	**30 579 306**	**5%**	**36%**	**13%**

Using the in-depth results of the two case study countries, Afghanistan and Sudan, we find evidence for specific HR and training activities that have been influenced by Global Fund investments. As shown in Table 5, as of December 2017, a total of 32 grants have been awarded to both countries with 7 grants allocated to HIV/AIDS, 11 grants for Malaria, 10 grants for TB, and 3 grants for HSS/RSSH across both countries, while 1 grant has been awarded jointly for all three disease categories (HIV/AIDS, TB, and malaria) in Afghanistan. The US$ 647 million and US$ 491 million in Sudan account for 29% and 30% of all budget and expenditure to the entire region respectively. The US$ 233 million and US$ 152 million in Afghanistan account for 11% and 9% of all budget and expenditure to the entire region respectively (Table 3). In Afghanistan, the principal recipients for the grants are evenly distributed between government and private/non-government organizations. In Sudan, almost all grants are administered by the United Nations Development Programme.

**Table 5:** Summary of Global Fund-financed grants in Afghanistan and Sudan

		Afghanistan	Sudan
**Grants**			
Number of HIV/AIDS grants		3	4
Number of malaria grants		7	4
Number of TB grants		6	4
Number of HSS grants		2	1
**Total number of grants**		19*	13
**Budgets (in millions US$)**			
HIV		24	177
Malaria		124	355
TB		44	98
HSS		41	17
**Total**		233	647
**Expenditure (in millions US$)**			
HIV		19	118
Malaria		79	295
TB		30	68
HSS		24	9
**Total**		152	491
**Type of principal recipients**			
Government		9	1
NGO/private		10	12

*HIV/AIDS* human immunodeficiency virus/acquired immunodeficiency syndrome, *TB* tuberculosis, *HSS* health systems strengthening, *NGO* non-governmental organization

*An additional grant was awarded jointly for HIV/AIDS, TB, and malaria

### Qualitative findings

In Afghanistan, as shown in Table 6, Global Fund financing has been instrumental in supporting both in-service and pre-service trainings with the aim of building the capacities of health sector personnel in the country. The beneficiaries of in-service trainings include national program officers, provincial program officers, health management information systems officers, or clinical staff, such as medical doctors, nurses, community health workers, community health supervisors, and lab technicians. One pre-service training program that the Global Fund invested heavily in is the Community Health Nursing Education program that provides a 2-year training for female community health nurses with a focus on health needs of rural populations, as well as specific diseases including HIV/AIDS, TB, and malaria. The graduates, who make formal commitments to serve their community for 3–5 years after graduation, are also involved in other relevant community health activities, such as home visits and supportive supervision of community health workers. Six hundred and seventy-three community nurses (a 97% completion rate) have graduated from this program, and a 2016 assessment showed an estimated 59% of program graduates had been deployed to public health facilities in their communities [20].

**Table 6:** Summary of Global Fund-supported HRH activities

		Afghanistan	Sudan
**Training**			
Type of training		In-service, pre-service	In-service, pre-service
Disease/HSS focus		HIV, TB, malaria, HSS	HIV, TB, malaria, HSS
Public/private health workers trained		Public and private	Public and private
**Human resource activities**			
Hiring/contracting/recruitment		Program coordinators and staff	Program coordinators and staff
Innovative financing used to supplement salaries		Top-up for clinical staff (physicians), lab technicians, IDP camp outreach workers	Top-up for clinical staff

*HRH* human resources for health, *TB* tuberculosis, *IDP* internally displaced persons, *HSS* health systems strengthening

Similarly, in Sudan, Global Fund has supported both pre-service and in-service training. Global Fund provided some funding for infrastructure (e.g., vehicles and rehabilitation of buildings) to the Academy of Health Sciences, which was established by the Ministry of Health in 2005 to train health professionals, including nurses and community health workers. An example of pre-service training though this academy is the primary health care expansion program targeted at producing primary health care cadres, namely community health worker/volunteers, through a 9-month training program in integrated primary health care delivery. Global Fund financing has also played a role in the functioning of Continuous Professional Development (CPD) centers which were established to provide in-service training through short courses for all levels and disciplines of health cadres in alignment with the country’s needs. An example of a CPD in-service training is a 45-day bridging course for medical assistants to receive on-the-job training in integrated care provision. Support has also been provided for various in-service trainings for health workers involved in HIV/AIDS, TB, and malaria control programs.

The case study results with regard to hiring, contracting, recruitment, and compensation of health care workers in Afghanistan and Sudan indicate that the Global Fund does not provide direct salary support for health workers. This is due to Global Fund and government policies, as well as concerns about sustainability and health worker motivation. Hence, Global Fund’s influence in this regard in Afghanistan is mainly through the payment of incentives or top-ups for health workers, such as health workers who have worked in TB treatment and prevention and outreach workers at internally displaced peoples’ camps. According to the public health ministry’s policies, funds for incentives could not exceed 10% of the payroll costs of the individual facility or 5% of the payroll costs of the grant or contract. Global Fund also supported top-up payments to health care workers in Sudan until 2016, but currently is not investing in such payments.

Regarding the level of coordination between Global Fund-supported HRH-related activities and national governments’ programming, in Afghanistan, in-service trainings funded by Global Fund are launched in close collaboration with the Ministry of Public Health. The planning, training materials, and implementation of these trainings are done by the national HIV/AIDS, TB, and malaria disease programs. There are noted gaps in information management related to tracking and keeping records on the number of trainees across agencies and donors. In Sudan, there is coordination between the government and donors for some activities. For example, the creation of “One Plan” by the Federal Ministry of Health is to ensure complementarity, harmonization, and reduction in duplication of donor-supported activities. Hence, Global Fund-supported activities are directed to identified areas of need that complement other donor-supported programs. In addition, relevant stakeholders discuss how to deploy the investments from the Global Fund to ensure alignment with the goals of the Ministry of Health and the needs of the country. For example, the discussion on how resources provided to the Academies of Health Sciences were to be utilized took place between the officials of the academy, the Ministry of Health, other relevant government agencies, and the Global Fund.

## Discussion

We find that approximately 27–28% of Global Fund’s 2003 to 2017 total budgets and expenditures are allocated to HRH in this region (as reported in Table 3 and Fig. 3). This is greater than the global average of 23% estimated by Bowser et al. (2014) as being allocated to HRH in their review of Global Fund investments across 138 recipient countries between 2003 and 2007 [7]. However, as shown above, the amount budgeted and expensed for HRH as a percent of total Global Fund financing has increased over time in the EMR, possibly reflecting the increased priority placed on health systems strengthening by the Global Fund within the three disease components of this study and separately through the HSS/RSSH component.

Our analysis of direct estimates of HRH budgets between 2015 and 2017 provides a more in-depth examination of budgetary allocations to those cost groupings that most “directly” support HRH in each country. The direct analysis suggests that only about a third of allocations to HRH (averages of 13% [direct HR] compared to 36% [total HR] between 2015 and 2017) directly impact local health workers. This is an especially important finding, as it helps provide a more accurate picture of HRH investments which could be overestimated when payment to employees of in-country contractors who manage the Global Fund grants are also included. These results are not meant to suggest that funding for grant and program management is not important, but to stress the importance of having an accurate measure of funds that directly support healthcare workers in Global Fund countries [10]. This level of disaggregation is only possible for the most recent years of budgeting (2015–2017) due to significant improvements in data collection and feedback between recipient countries and the Global Fund. In addition, a more disaggregated analysis is only available for budgetary data and not expenditures. Expenditure data would provide a more accurate depiction of spending on in-country HRH supporting activities.

Global Fund has supported pre-service and in-service trainings in both Afghanistan and Sudan. In Afghanistan, the pre-service training programs have been useful in increasing the number of health workers, as well as addressing the gender imbalance in HRH shortage. This is particularly important in this setting where cultural barriers prevent female patients from accessing health care from male health workers. In Sudan, Global Fund support for pre-service training has been channeled through funding of infrastructure needed to train health workers. Investment in pre-service training in both countries is noteworthy because previous research found that global health initiatives such as Global Fund’s HR investment is mainly focused on in-service training [8–10]. Noting the downward trend in nurse densities in both countries over time and more generally the low HRH densities in recipient countries (Table 2), a reduction in this imbalance between in-service and pre-service training investment can help begin to address critical health worker shortage issues. In-service training programs focused on integrating care also have potential to reduce fragmentation and improve delivery of care in these countries [21]. In addition, some of the training programs, such as the Academy of Health Sciences, demonstrate a degree of institutionalization whereby the establishment and implementation of a training center is driven by local officials while being supported by Global Fund investment. However, concerns remain about the continued quality and operation of these centers and programs at the end of Global Fund financing.

Human resource investment includes salary support in terms of top-ups and performance incentives, as the Global Fund does not directly pay health workers’ salaries in the two countries, except the program management staff at the national level. This might mitigate to some extent concerns related to sustainability, health worker motivation, and displacement found in previous studies [9, 12, 22]. However, it does not address the limited capacity of the health ministries to absorb and assimilate newly trained health workers into the public health sector. The “direct” HR budgetary allocations in Afghanistan (14%) and Sudan (10%) at the time of the study suggest that there is room to increase these allocations in order to scale up health workforce to achieve a sustainable health workforce for the future, including investment in production capacities. The low health workforce densities, coupled with limited employment capacities and lack of incentives to retain health care workers, implies the need to consider increasing investment in health workforce to ensure the delivery of services, commodities, and building health workforce as countries graduate from Global Fund's support.

There is evidence of close coordination between Global Fund investments in HRH and relevant departments and stakeholders in Sudan. The creation of the “One Plan” initiative is an example of a proactive approach to ensuring coordination and harmonization between external donors, local implementers and the Ministry of Health. This approach can be adapted to other contextual settings as a potential way of addressing the issue of poor coordination among stakeholders reported by other studies (e.g. [7, 9, 11]). However, we find a gap in the level of coordination between grants, government agencies, and principal recipients (grant managers) over time. This gap may only be effectively bridged by the recipient country governments ensuring that knowledge, lessons, and information from a grant are carried over to the next grant and thus build institutional memory. While not directly analyzed as part of the study, having two HSS/RSSH grants in Afghanistan and one HSS/RSSH grant in Sudan (three HSS/RSSH grants in total across both countries) suggests a focus on system strengthening in addition to disease-specific programs.

This study provides new insights into EMR Global Fund investments from its onset until 2017 and also serves as an update to a previous study that used similar data from 2002 to 2010 [7]. There are, however, some limitations that highlight the challenges and complexity of tracking large-scale investments such as those described in this study and understanding their impact on recipient countries. First, available data on expenditure were not disaggregated to a level that linked monetary amounts to specific training and HR activities. Hence, the high-level aggregates of the proportion of HRH budget and expenditure allocations from 2003 to 2017 may be overestimated, as they include line items such as salaries of organizations managing the grants in recipient countries.

Another, data-related limitation is that we are not able to evaluate the impact of Global Fund investment on health outcomes in recipient countries, which would be a potential next step in further elucidating the effect of Global Fund HRH investments in recipient countries. In addition, it was only possible to collect in-depth data on two country case studies. Additional case studies could have enhanced the qualitative nature of the study.

## Conclusion

This study presents new evidence on the magnitude and composition of Global Fund’s HRH investments and the HRH-related outputs in the EMR. Analysis of high-level aggregate data between 2003 and 2017 finds that about a third of Global Fund budget and expenditure goes to HRH in this region according to the overall analysis using Global Fund-defined budget lines, while analysis of more recent detailed data between 2015 and 2017 suggests a more conservative estimate of about 13%. In addition, Global Fund investments are being used to support outputs such as pre-service and in-service training as well as salary support such as top-ups and performance incentives. There appears to be clear examples of Global Fund investments contributing to sustainable and institutionalized HRH outputs and some donor coordination in the two case countries. These findings suggest a need for improved information management systems to better track HRH expenditure and key HRH outputs. HRH remains a key issue in strengthening the health system of LMICs and even more so in areas of the globe prone to tension and conflict. Considering the unique HRH challenges in this region, this study indicates a need to continue investments in HRH and analyses of this topic.

## Supplementary information

**Additional file 1: Table S1.** Summary of Global Fund HRH activities of four recent grants in Afghanistan (2015-2017). **Table S2.** Comparison of human resources and training as percentage of total budget and expenditure pre-post 2007.

## Data Availability

The datasets used and/or analyzed during the current study are available from the corresponding author on reasonable request.

## Abbreviations

CPD: Continuous Professional Development
EFR: Enhanced Financial Reporting
EMR: Eastern Mediterranean Region
HIV/AIDS: Human immunodeficiency virus/acquired immunodeficiency syndrome
HR: Human resources
HRH: Human resources for health
HSS/RSSH: Health systems strengthening/resilient and sustainable systems for health
LMICs: Low- and middle-income countries
TB: Tuberculosis
TRC: Travel-related costs
