# Supplementary material for: Global Fund financing and human resources for health investments in the Eastern Mediterranean Region
Source: Hum Resour Health. 2020 Jul 8;18:48. doi: 10.1186/s12960-020-00483-x (PMC7341639; doi:10.1186/s12960-020-00483-x)
Supplement: Supplementary file 1 — Additional file 1: Table S1. Summary of Global Fund HRH activities of four recent grants in Afghanistan (2015-2017). Table S2. Comparison of human resources and training as percentage of total budget and expenditure pre-post 2007. [file 12960_2020_483_MOESM1_ESM.docx]

# Appendix

**Appendix Table 1: Summary of Global Fund HRH activities of four recent grants in Afghanistan (2015-2017)**

|  |  | **AFG-S-UNPD** | **AFG-T-UNDP** | **AFG-M-UNDP** | **AFG-H-UNDP** |
| --- | --- | --- | --- | --- | --- |
| **Training** | | | |  |  |
|  | Type of training | In-service, pre-service | In-service | In-service | In-service |
|  | Disease/HSS focus | HSS | TB | Malaria | HIV/AIDS |
|  | Public/Private health workers trained | Public and Private | Public and Private | Public and Private | Public and Private |
| **Human resource activities** | | | |  |  |
|  | Hiring/ contracting/recruitment | Management staff (Operational staff: admin/finance/procurement; and Program staff: e.g. Provincial Coordinator, Lab Coordinator etc.) | Management staff (Operational staff: admin/finance/procurement; and Program staff: e.g. Provincial Coordinator, Lab Coordinator etc.) | Management staff (Operational staff: admin/finance/procurement; and Program staff: e.g. Provincial Coordinator, Lab Coordinator etc.) | Management staff (Operational staff: admin/finance/procurement; and Program staff: e.g. Provincial Coordinator, Lab Coordinator etc.) |
|  | Innovative financing used to supplement salaries | Top-up for management staff | Top-up for management and clinical staff | Top-up for management staff | Top-up for management staff |

Notes: HRH denotes human resources for health; AFG denotes Afghanistan; TB denotes tuberculosis; IDP denotes internally displaced persons; HSS denotes health systems strengthening; HIV/AIDS denotes human immunodeficiency virus/acquired immunodeficiency syndrome; UNDP denotes United Nations Development Programme,. The UNDP Global Fund grants in Afghanistan are for Health Systems (S), TB (T), Malaria (M), and HIV/AIDS (H).

**Appendix Table 2: Comparison of human resources and training as**

**percentage of total budget and expenditure pre-post 2007**

|  | 2003-2007 | | 2008-2017 | |
| --- | --- | --- | --- | --- |
| **Country** | Budget | Expenditure | Budget | Expenditure |
| Afghanistan | 23% | 27% | 32% | 34% |
| Djibouti | 20% | 21% | 28% | 34% |
| Egypt | 28% | 35% | 30% | 39% |
| Iran (Islamic Republic) | 51% | 41% | 34% | 34% |
| Iraq | - | - | 26% | 27% |
| Jordan | 18% | 17% | 31% | 38% |
| Morocco | 38% | 35% | 31% | 34% |
| Pakistan | 23% | 23% | 29% | 30% |
| Somalia | 23% | 23% | 32% | 35% |
| Sudan | 6% | 18% | 23% | 21% |
| Syrian Arab Republic | 22% | 27% | 22% | 25% |
| Tunisia | 28% | 49% | 36% | 41% |
| Yemen | 21% | 20% | 14% | 15% |
| **Average** | **21%** | **23%** | **28%** | **28%** |
